# Vitamin A Transport and the Transmembrane Pore in the Cell-Surface Receptor for Plasma Retinol Binding Protein

**DOI:** 10.1371/journal.pone.0073838

**Published:** 2013-11-01

**Authors:** Ming Zhong, Riki Kawaguchi, Mariam Ter-Stepanian, Miki Kassai, Hui Sun

**Affiliations:** Department of Physiology, Jules Stein Eye Institute, and Howard Hughes Medical Institute, David Geffen School of Medicine, University of California, Los Angeles, California, United States of America; National Eye Institute, United States of America

## Abstract

Vitamin A and its derivatives (retinoids) play diverse and crucial functions from embryogenesis to adulthood and are used as therapeutic agents in human medicine for eye and skin diseases, infections and cancer. Plasma retinol binding protein (RBP) is the principal and specific vitamin A carrier in the blood and binds vitamin A at 1∶1 ratio. STRA6 is the high-affinity membrane receptor for RBP and mediates cellular vitamin A uptake. STRA6 null mice have severely depleted vitamin A reserves for vision and consequently have vision loss, even under vitamin A sufficient conditions. STRA6 null humans have a wide range of severe pathological phenotypes in many organs including the eye, brain, heart and lung. Known membrane transport mechanisms involve transmembrane pores that regulate the transport of the substrate (e.g., the gating of ion channels). STRA6 represents a new type of membrane receptor. How this receptor interacts with its transport substrate vitamin A and the functions of its nine transmembrane domains are still completely unknown. These questions are critical to understanding the molecular basis of STRA6′s activities and its regulation. We employ acute chemical modification to introduce chemical side chains to STRA6 in a site-specific manner. We found that modifications with specific chemicals at specific positions in or near the transmembrane domains of this receptor can almost completely suppress its vitamin A transport activity. These experiments provide the first evidence for the existence of a transmembrane pore, analogous to the pore of ion channels, for this new type of cell-surface receptor.

## Introduction

Vitamin A is a multifunctional chemical essential for human life. In addition to its function as the light sensor in vision [1], [2], [3], [4], [5], [6], vitamin A plays critical roles in almost all human organs and regulates cell growth and differentiation from embryogenesis to adulthood [7], [8]. An imbalance in retinoid homeostasis is associated with a variety of human diseases including visual disorders [4], infectious diseases [9], [10], [11], neurological disorders [12], [13], teratogenicity [14], cancer [15], and skin diseases [16], [17], [18]. Precise and specific transport of vitamin A is important not only because of its essential biological functions but also the toxicity associated with random diffusion of vitamin A or its derivatives [14], [19], [20]. Plasma retinol-binding protein (RBP) is the primary physiological transport vehicle for vitamin A in the blood [21], [22], [23], [24], [25], [26]. The crystal structures of RBP and its complex with transthyretin have been determined [27], [28]. It was first proposed in the 1970s that a cell-surface receptor mediates vitamin A uptake from RBP [29], [30], [31]. This receptor has been identified as a multitransmembrane domain protein STRA6 [32]. STRA6 was originally known as a cancer cell-surface protein [33], [34]. STRA6 binds to RBP with high affinity and mediates cellular uptake of vitamin A [32].

STRA6 mutations are associated with severe pathological phenotypes in multiple human organs including the eye, brain, lung and heart [35], [36]. Animal studies demonstrated that loss of STRA6 suppresses vitamin A uptake in zebrafish [37] and mice [38]. STRA6 is responsible for about 95% of vitamin A uptake for vision under vitamin A sufficient conditions [38]. What's responsible for the STRA6-independent 5%? It has been known since the 1970s that small intestine-secreted lipoprotein particle that contains retinyl ester is the predominant pathway to transport vitamin A independent of RBP under vitamin A sufficient or excessive conditions [19], [23]. Under vitamin A deficient conditions that mimic natural environments, RBP knockout is embryonic lethal due to the inability of the retinyl ester pathway to mobilize vitamin A stored in the liver [39], [40]. Consistent with the role of STRA6 as the RBP receptor, STRA6 knockout mice [38], like RBP knockout mice [24], [41], have mild systemic phenotypes and primarily vision defects under vitamin A sufficient conditions. Why does the loss of STRA6 in mice cause severe phenotypes only in the eye, while the loss of STRA6 in humans causes severe systemic phenotypes in multiple organs? One well known species difference between humans and mice is the high sensitivity of humans to the toxicity associated RBP-independent pathways such as the retinyl ester bound to liporoteins [19]. Rodents are about 100 times more resistant to the toxicity associated with random retinoid diffusion than humans [42]. Despite the species differences, a common theme has emerged. The eye is the organ most sensitive to vitamin A deficiency, the loss of RBP or the loss of STRA6 in both humans and mice. A complete null of RBP has never been found in human populations, but a partial loss of human RBP function (a mutant that still binds retinol and TTR) also leads to vision-specific phenotypes [43], [44].

STRA6 is a new type of cell-surface receptor [45]. The transmembrane topology of STRA6 was previously unknown. One study systematically determined that STRA6 has 9 transmembrane domains, not the 10 or 11 transmembrane domains predicted by computer software [46]. An essential RBP-binding domain was revealed as a result of an unbiased screening of STRA6 mutants [47]. STRA6-mediated vitamin A uptake from RBP does not depend on the endocytosis of the RBP protein [32]. But how does STRA6 transport vitamin A from extracellular RBP to inside the cell? STRA6′s vitamin A uptake is distinct from known mechanisms such as active transport (which depends on cellular energy), channels and facilitated transport (which depend on the electrochemical gradient of the free substrate). STRA6′s substrate vitamin A is only provided one at a time through RBP and is not free but bound with high affinity to RBP. The first clue to STRA6′s mechanism was the finding that lecithin retinol acyltransferase (LRAT) [32], [37], [48] or cellular retinol binding protein I (CRBP-I) [49] stimulates STRA6′s vitamin A uptake activity. By distinguishing holo-RBP bound to cell-surface STRA6 and true intracellular uptake, it was found that STRA6-mediated vitamin A uptake is tightly coupled to LRAT or CRBP-I [49]. In the absence of LRAT and CRBP-I, STRA6 catalyzes retinol release from holo-RBP [49], [50] and retinol loading into apo-RBP [37], [49], [50]. STRA6-catalyzed retinol release activity is largely responsible for its ability to take up vitamin A from RBP, which remains outside of the cell [49]. The ability of STRA6-mediated retinol loading to counteract STRA6′s retinol release is responsible for STRA6′s coupling to LRAT and CRBP-I, which can inhibit the loading activity [49].

One of the most critical questions in any membrane transport mechanism is how the transport substrate interacts with the transport machinery. For example, investigation of the pores of the potassium channels led to landmark discoveries on ion channel mechanisms [51], [52], [53], [54], [55], [56], [57]. How STRA6 interacts with its transport substrate vitamin A is completely unknown. Since vitamin A can freely diffuse through membranes, two possibilities exist for how vitamin A released from RBP crosses the plasma membrane: 1) retinol travels within a specific “pore” in STRA6′s transmembrane helices; 2) retinol diffuses randomly through the plasma membrane after it is released from RBP. Unlike some channels or transporters, there is also no known chemical or toxin that can block vitamin A transport by the RBP receptor. In addition, unlike the substrates of ion channels, vitamin A has no charge. Changing the charge of a key residue in the transport pathway can be sufficient to block ion channels (e.g., due to charge repulsion), but a similar kind of change may not affect the transport of vitamin A due to its lack of charge. In this study, we overcome these difficulties using complementary techniques to provide the first experimental and structural evidence that there exists a transmembrane pore that interacts with vitamin A in this new type of cell-surface receptor. This study also provides the first demonstration that chemical modification can almost completely inhibit vitamin A transport by the RBP receptor. The inhibitory effects are both highly site specific and chemical modification specific.

## Materials and Methods

### Production of holo-RBP, apo-RBP, ^3^H-retinol/RBP, EGFP-CRBP-I, and STRA6 mutants

Holo-RBP was produced, refolded, and purified using HPLC as previously described [32]. Apo-RBP was prepared from holo-RBP by extracting retinol as described [58]. His-EGFP-CRBP-I was expressed in COS-1 cells. Cell pellets were lysed with 1% Triton in PBS and His-EGFP-CRBP-I was purified on Ni-NTA resin (*Qiagen*). Random STRA6 mutants were produced as previously described [47]. Site directed mutagenesis was carried out with overlap extension PCR. The final constructs were sequenced to rule out spurious mutations.

### Vitamin A uptake assay based on ^3^H-retinol/RBP

Radioactive all-trans retinol [11, 12-^3^H(N)] was purchased from *PerkinElmer*. ^3^H-retinol/RBP was prepared as described [32]. For live-cell vitamin A uptake assays, COS-1 cells were chosen because they adhere to the cell culture dish strongly and does not detach during repeated washes and media changes. The other commonly used cell type HEK293 cell is not useful for this assay because of its weak adherence that can cause complete cell loss after a few washes. Cell transfection has been described previously [32]. Cells were washed once with Hank's Balanced Salt Solution (HBSS) and grown in serum free medium (SFM) overnight before the assay. The overnight SFM incubation allowed the dissociation of RBP (in the serum used in cell culture) from STRA6 on the cell surface, as this association can block the binding of ^3^H-retinol/RBP to STRA6. On the day of the assay, ^3^H-retinol/RBP in fresh SFM was added at time 0. After incubation at 37°C for 1 hour, the cells were washed with HBSS and solubilized in 1% Triton X-100 in PBS. ^3^H-retinol remaining in the cells was measured with a scintillation counter.

### Cell-free assay for ^3^H-retinol uptake

Membrane preparation and the cell-free assay for vitamin A uptake was performed similarly as described previously [32]. Membrane preparations were treated with 10 mM methanethiosulfonate ethylammonium-biotin (MTSEA-biotin) for 30 min at room temperature and were then washed to remove the chemical before the assay. To study the coupling of STRA6 to LRAT, membranes prepared from cells expressing STRA6 and LRAT were mixed together and incubated with ^3^H-retinol/RBP in PBS for 1 hour at 37°C. The reactions were stopped by passing through filters in MultiScreen HTS filter plate (*Millipore*) and washing with PBS to remove all free ^3^H-retinol/RBP. Radioactivity associated with the filter was measured with a scintillation counter.

### HPLC-based assays of vitamin A uptake

For MTSEA-biotin treatment, cells were incubated with 10 mM MTSEA-biotin at 37°C for 30 min and washed with HBSS. After incubation for 4 hours with 25% normal human serum diluted in SFM, cells were harvested and extracted 3 times with hexane. The retinyl-ester contents were quantified with HPLC. For cell-free HPLC assay, the membrane fraction from cells expressing STRA6 or STRA6 mutants was incubated with 10 mM MTSEA-biotin at room temperature for 30 min and washed thoroughly to remove any residual modification reagent. After modification, STRA6-expressing membranes and lecithin retinol acyltransferase (LRAT)-expressing membranes were mixed together and incubated with 1 µM holo-RBP in PBS for 1 hour at 37°C. The reactions were stopped by pelleting down membrane at 4°C. The membrane pellet was extracted with hexane and analyzed with HPLC for retinyl ester. Retinyl ester was separated using a Zorbax Eclipse EDB-C18 column (*Agilent*). The ethyl acetate concentration was increased from 0 to 100% against methanol during the 10 min run, followed by a 5 min wash with 100% ethyl acetate. Retinyl ester was detected by absorbance at 325 nm.

### Real-time analysis of STRA6-catalyzed retinol release from holo-RBP

STRA6′s activity in mediating retinol release was studied as described [59]. Briefly, a Microfluor-2 plate (*Thermo*) was pre-coated overnight with Blocker Casein (*Pierce*) and washed once with PBS before addition of membrane suspension to prevent nonspecific sticking of holo-RBP to the plastic wall. Stocks of 1 M 2-hydroxyethyl methanethiosulfonate (MTSEH), 3-trimethylammoniumpropyl methanethiosulfonate (MTSPT), 4-sulfonatobutyl methanethiosulfonate (MTSBS) or MTSEA-biotin in DMSO were added to corresponding wells with membrane suspension to achieve a final concentration of 10 mM. Real-time monitoring of retinol fluorescence was measured using a fluorescent microplate reader POLARstar Omega (*BMG LABTECH*) with the excitation filter 320EX and the emission filter 460-10. The signal from each time points is the average of 10 measurements. Samples were shaken for 10 sec at 500 rpm using double-orbital shaking before each measurement. The signals before the addition of holo-RBP at 0 min are considered background signals and were subtracted from the final fluorescence signals at different time points.

### Real-time analysis of STRA6-catalyzed retinol transport from RBP to CRBP-I using fluorescence resonance transfer (FRET)

The establishment of retinol and EGFP as a FRET pair and the EGFP-CRBP-I fusion protein were described previously [49]. Holo-RBP was added to EGFP-CRBP-I premixed with membranes to initiate the reactions. Retinol-EGFP FRET was measured using simultaneous dual emission optics in POLARstar Omega with the excitation filter 320ex and the emission filters 460-10 and 510-10. The background signal of each reaction was measured before holo-RBP was added at 0 min. The signal from each time point was the average of 10 measurements. After all the measurements were done, the FRET signals were calculated using the equation [(510_t_-510_b_)/(460_t_-460_b_)], where 510_t_, 510_b_, 460_t_, and 460_b_ represent emissions at 510 nm after initiation of the reaction (t = time point), at 510 nm before holo-RBP is added (b = background), at 460 nm after initiation of the reaction (t = time point), and at 460 nm before holo-RBP is added (b = background), respectively.

### Statistical analysis

Raw data was processed in Excel and then imported to RStudio (version 0.97.318). One-way ANOVA and post-hoc analysis were performed using the Multcomp package (http://cran.r-project.org/web/packages/multcomp/index.html).

## Results

### Large-scale mutagenesis to reveal STRA6 mutants defective in vitamin A uptake

We first used random mutagenesis to identify STRA6 mutants that bind RBP but fail to transport vitamin A. Random mutagenesis has been very successful as an unbiased technique to elucidate biological mechanisms [60], [61] and has also been used to study transport mechanisms such as the transmembrane pores of potassium channels [62], [63], [64]. The most efficient way to do large-scale mutant screen is to use a genetic model such as yeast [62], [63], [64]. However, it is not feasible to design a yeast survival assay to study the transport of vitamin A. Therefore, we manually produced and functionally tested a large number of random mutants as an unbiased strategy to study STRA6′s structure and function. We did successfully identify a large number of STRA6 mutants that have lost the vitamin A uptake activity (Figures S1, S2, S3, and S4). However, most defective STRA6 mutants lose their vitamin A uptake activity because they do not express on the cell surface. We did not find a mutant that still binds RBP but does not transport vitamin A. It is possible that the chance of a random mutation to block vitamin A transport is very low. Another possible explanation is that it is difficult to block vitamin A transport by mutation alone because vitamin A is a hydrophobic chemical that has no charge.

### Acute chemical modification of STRA6

In addition to mutations, exogenous reagents have been used to study membrane transport. For example, blocking the pore of a potassium channel using a toxin revealed the structural basis of its substrate transport [52]. Because no toxin or related reagent is known to block vitamin A transport by the RBP receptor, we employed an alternative strategy of chemical modification of introduced cysteine residues [65]. The advantage of this strategy is that cysteine mutations in the transmembrane regions tend not to disturb protein folding, and we can start with a functional protein before chemical modification. In addition, chemical modification makes it possible to introduce types of side chains not possible by mutagenesis alone. One difficulty of using this strategy is that STRA6 has 14 natural cysteines and it is not practical to produce a cysteine-less “wild-type” STRA6. Our strategy was to test if an introduced cysteine conferred any additional property to STRA6 after chemical modification.

**Figure 1:**
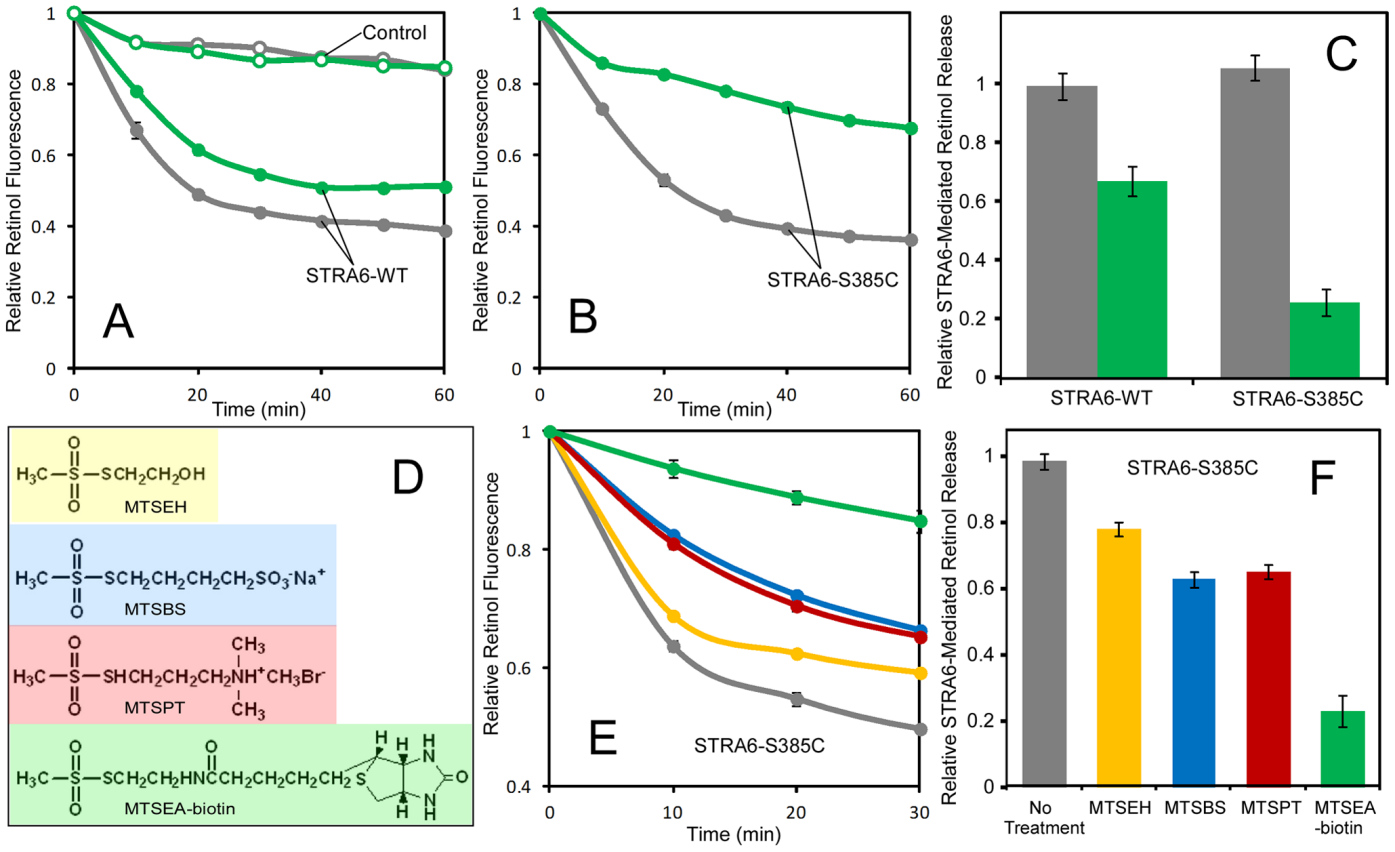
Effects of acute chemical modification on STRA6-catalyzed retinol release from holo-RBP. STRA6-catalzyed retinol release from holo-RBP causes a decline in retinol fluorescence. In all experiments, holo-RBP was added at 0 min. **A and B.** Effects of MTSEA-biotin treatment on the retinol release activity of STRA6-WT and STRA6-S385C, respectively. Grey traces, no treatment. Green traces, MTSEA-biotin treatment. Control membrane without STRA6 was used as the negative control in A (open circles). **C.** Comparison of the effects of MTSEA-biotin on STRA6-WT and STRA6-S385C. Activity of the untreated reaction for STRA6-WT is defined as 1. Grey bars, no treatment. Green bars, MTSEA-biotin treatment. **D.** Structures of MTSEH, MTSBS, MTSPT and MTSEA-biotin. **E.** Comparison of effects of MTSEH, MTSBS, MTSPT and MTSEA-biotin of retinol transport activity of S385C. The color of the trace in E matches the color of the chemical in D. Grey trace, no treatment. **F.** Quantitation of retinol release activity of experiments in E. Activity of the untreated reaction is defined as 1.

We chose reagents that differ in charge and size for cysteine modification (MTSEH, MTSBS, MTSPT, and MTSEA-biotin) (Figure 1). Of these four chemicals, MTSEH has the smallest size, MTSPT is positively charged, MTSBS is negatively charged, and MTSEA-biotin has the largest size. It was known previously that STRA6 is capable of catalyzing retinol release from holo-RBP [49]. This activity is largely responsible for endocytosis-independent uptake of vitamin A from holo-RBP. Using real-time analysis of STRA6-catalyzed retinol release and these chemicals, we initially studied residues within the seventh transmembrane domain and identified position S385 as highly sensitive to chemical modification (Figure 1). Without modification, STRA6-S385C has similar activity compared to the wild-type STRA6 (Figures 1A–1C). However, modification by MTSEA-biotin strongly suppresses STRA6-catalyzed retinol release for STRA6-S385C, although it also affects the wild-type protein to a much lesser extent (Figures 1A–1C). We also found that the inhibitory effect on STRA6-catalyzed retinol release is correlated only with the size of chemicals (Figures 1D–1F). MTSEH, which has the smallest size, has the smallest effect on blocking vitamin A release, while MTEA-biotin, which has largest size, shows strongest inhibition of STRA6-catalyzed retinol release. Positive or negative charge on the reagent has no additional effect on blocking vitamin A release, consistent with the fact that the substrate vitamin A has no charge.

### Modification of STRA6-S385C by MTSEA-biotin blocks transport of vitamin A from RBP to CRBP-I

**Figure 2:**
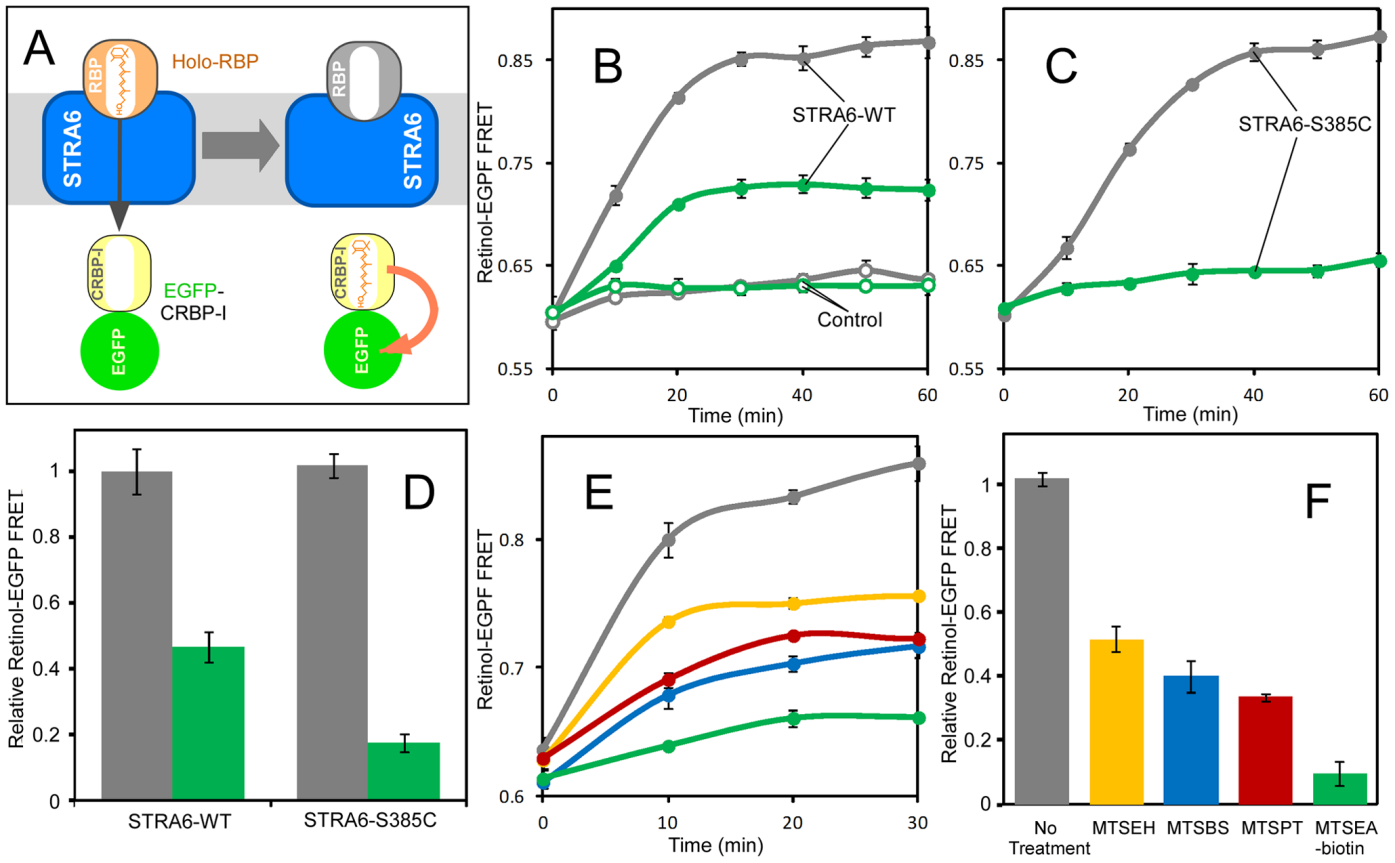
Real-time analysis of STRA6-catalyzed transport of retinol from holo-RBP to EGFP-CRBP-I. **A.** Schematic diagram of the use of retinol-EGFP FRET to monitor the transport of retinol from holo-RBP to EGFP-CRBP-I. FRET is indicated by an orange arrow. **B and C.** Effects of MTSEA-biotin treatment on the retinol transport by STRA6 WT and STRA6-S385C, respectively. Grey traces, no treatment. Green traces, MTSEA-biotin treatment. Control membrane without STRA6 was used as the negative control in B (open circles). **D.** Comparison of the effects of MTSEA-biotin on STRA6-WT and STRA6-S385C at 60 min. The signal of the untreated reaction for STRA6-WT is defined as 1. Grey bars, no treatment. Green bars, MTSEA-biotin treatment. **E.** Comparison of effects of MTSEH, MTSBS, MTSPT and MTSEA-biotin of retinol transport activity of STRA6-S385C. **F.** Quantitation of the FRET signals from experiments in E. The signal of the untreated reaction is defined as 1.

We next tested whether STRA6-mediated retinol transport from holo-RBP to CRBP-I can be blocked by acute modification of STRA6. To study STRA6-mediated retinol transport from holo-RBP to CRBP-I, we previously established retinol-EGFP as a new fluorescence resonance energy transfer (FRET) pair [49]. This transport activity can be monitored in real time by monitoring FRET between retinol and EGFP, which is fused to CRBP-I as a fusion protein (Figures 2A and 2B). The control reaction without STRA6 demonstrated the STRA6-dependence of the FRET signal (Figure 2B). Using this FRET-based technique, we again observed strong inhibition of STRA6-mediated transport of retinol from RBP to CRBP-I by chemical modification of STRA6-S385C (Figure 2). The transport of retinol is almost completely suppressed by the modification using MTSEA-biotin. The inhibitory effects of the reagents are correlated with their sizes (Figures 2E and 2F).

### Modification of STRA6-V320C or STRA6-S385C by MTSEA-biotin suppresses vitamin A transport to LRAT

**Figure 3:**
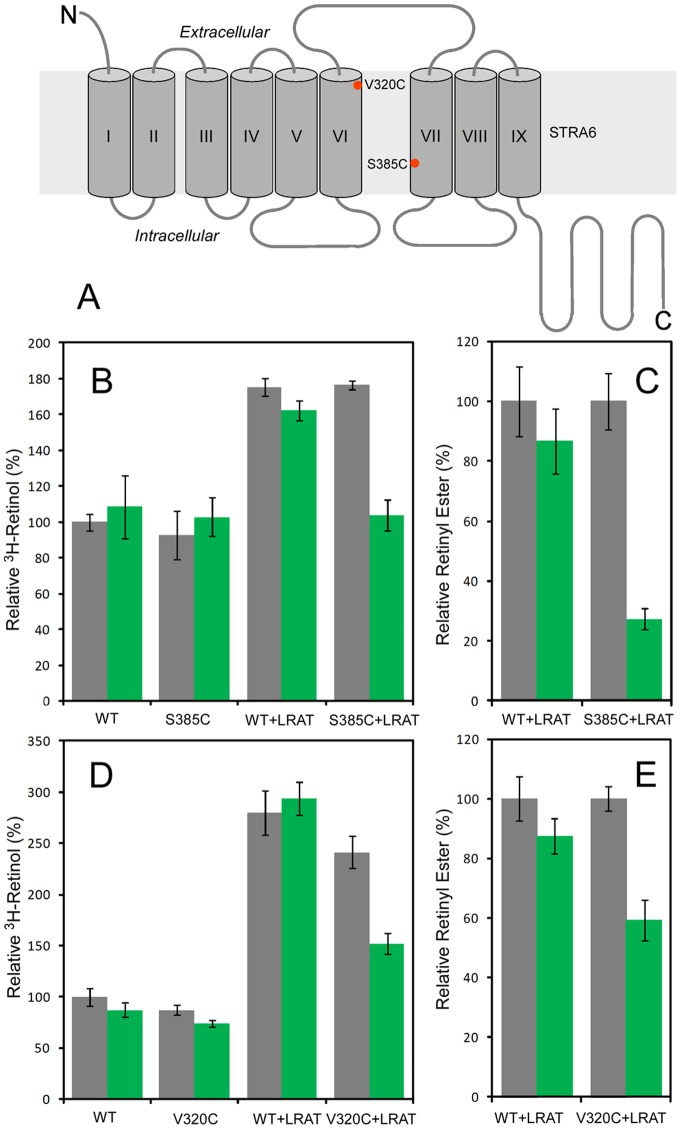
Effect of MTSEA-biotin modification on STRA6-mediated vitamin A uptake as measured by ^3^H-retinol uptake from ^3^H-retinol/RBP and HPLC analysis of retinyl esters. **A.** Positions of V320C and S385C in the transmembrane topology model of STRA6. Because MTSEA-biotin is membrane impermeable, the location of S385C close to the intracellular side of STRA6 makes it inaccessible to MTSEA-biotin from the extracellular side, while V320C is positioned on the boundary of membrane helix VI and extracellular domain and can be accessed by MTSEA-biotin from the extracellular side. **B and C.** Cell-free vitamin A uptake assays comparing the effect of MTSEA-biotin treatment on STRA6-WT (WT) and STRA6-S385C (S385C). B is ^3^H-retinol uptake assay from ^3^H-retinol/RBP. C is HPLC-based retinyl ester analysis of retinol uptake from holo-RBP. **D and E.** Live-cell vitamin A uptake assays comparing the effect of MTSEA-biotin treatment on STRA6-WT (WT) and STRA6-V320C (V320C). D is ^3^H-retinol uptake assay from ^3^H-retinol/RBP. E is HPLC-based retinyl ester analysis of retinol uptake from holo-RBP. In B and D, the amount of ^3^H-retinol associated with STRA6-WT without modification is defined as 100%. This activity reflects the binding of ^3^H-retinol/RBP to STRA6. In C and E, the activity of STRA6-WT with LRAT as measured by retinyl ester amount is defined as 100%. Grey bars, no treatment. Green bars, MTSEA-biotin treatment.

In addition to CRBP-I, LRAT is also known to couple to STRA6-mediated vitamin A uptake from holo-RBP. We next studied the effect of chemical modification on STRA6-mediated vitamin A uptake using both ^3^H-retinol-based vitamin A uptake assay and HPLC-based analysis of retinyl ester formation as a result of the coupling of STRA6-mediated vitamin A uptake to LRAT [32], [37], [48] (Figure 3). ^3^H-retinol/RBP can reveal both RBP's binding to STRA6 and STRA6′s coupling to LRAT, because ^3^H-retinol associated with STRA6 without LRAT largely reflects ^3^H-retinol/RBP bound to STRA6 [49]. In contrast, STRA6′s activity in the presence of LRAT reflects the coupling of STRA6 to LRAT [49].

Since MTSEA-biotin is membrane impermeable, we used a previously established cell-free vitamin A uptake assay [32] to test S385, a position located close to the cytoplasmic side (Figure 3A). This assay is based on the fact that both STRA6 and LRAT are membrane proteins and that retinyl ester produced by LRAT is associated with the membrane [32]. In the ^3^H-retinol based assay, we found that binding of ^3^H-retinol/RBP to STRA6-S385C is not affected by MTSEA-biotin modification, but STRA6-S385C's coupling to LRAT is highly suppressed by MTSEA-biotin modification (Figure 3B). In the HPLC-based analysis, the coupling of STRA6-mediated vitamin A uptake to LRAT to generate retinyl esters is also highly suppressed by MTSEA-biotin modification for STRA6-S385C (Figure 3C). We also found that a residue close to the extracellular side (V320) is sensitive to chemical modification to suppress STRA6′s vitamin A transport activity. Its location close to the extracellular side (Figure 3A) makes it possible to apply MTSEA-biotin treatment in live cells. We performed both ^3^H-retinol uptake assay from ^3^H-retinol/RBP (Figure 3D) and HPLC-based analysis of retinyl ester formation (Figure 3E). In both assays, MTSEA-biotin treatment highly suppresses the coupling of STRA6-V320C to LRAT (Figures 3D and 3E). These complementary experiments demonstrate that chemical modification of these key positions in STRA6 can block its vitamin A transport and its coupling to LRAT to produce retinyl esters.

### Locations of key positions in STRA6

**Figure 4:**
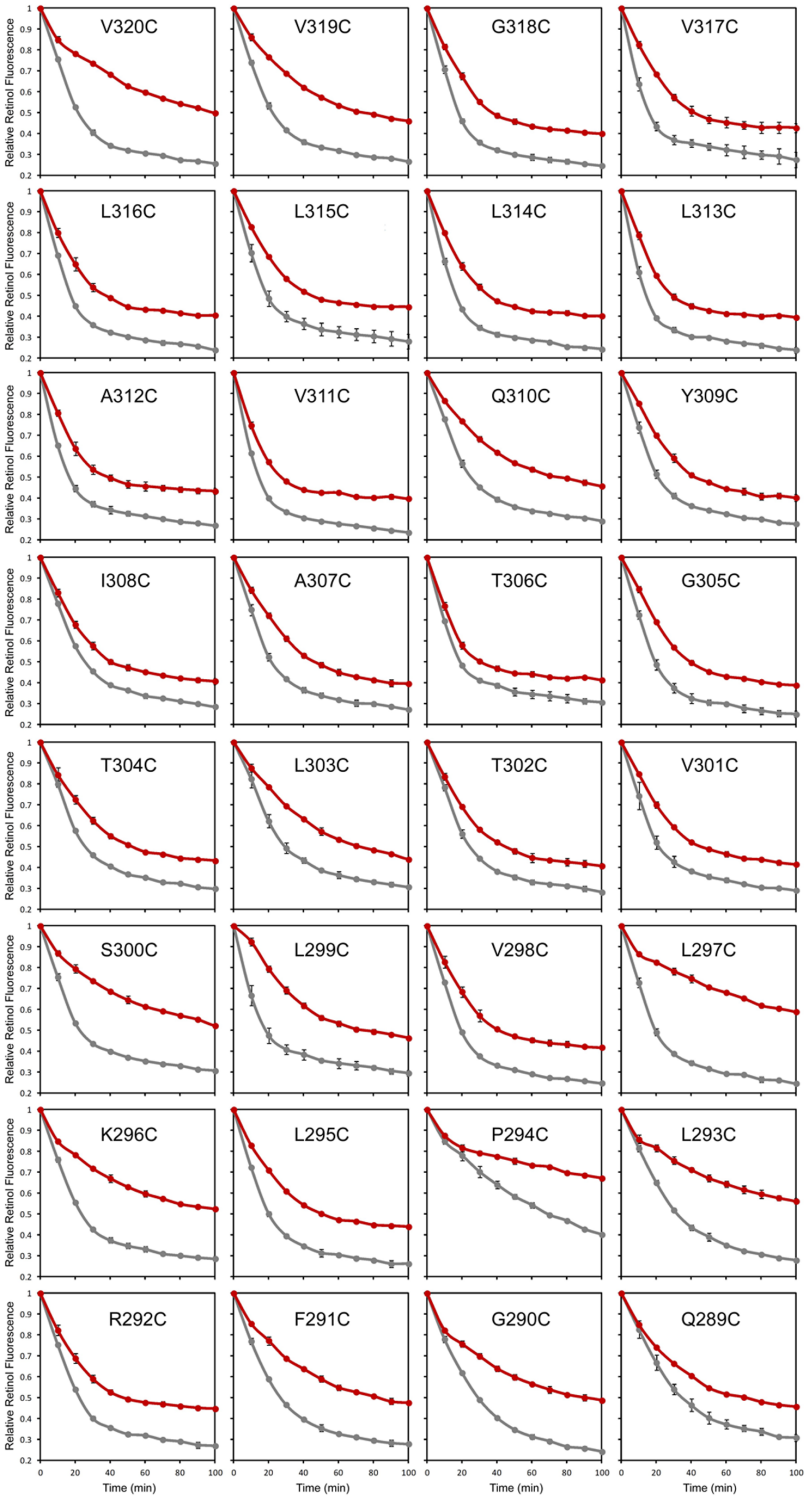
Scanning the sixth transmembrane helix of STRA6 for positions that enhance the sensitivity of STRA6 to MTSEA-biotin modification. Thirty two residues in or near the sixth transmembrane domain of STRA6 were each changed to cysteine. Each mutant was tested for STRA6-catalyzed retinol release from holo-RBP. Grey traces, no modification. Red traces, MTSEA-biotin modification. All experiments were done in triplicate.

**Figure 5:**
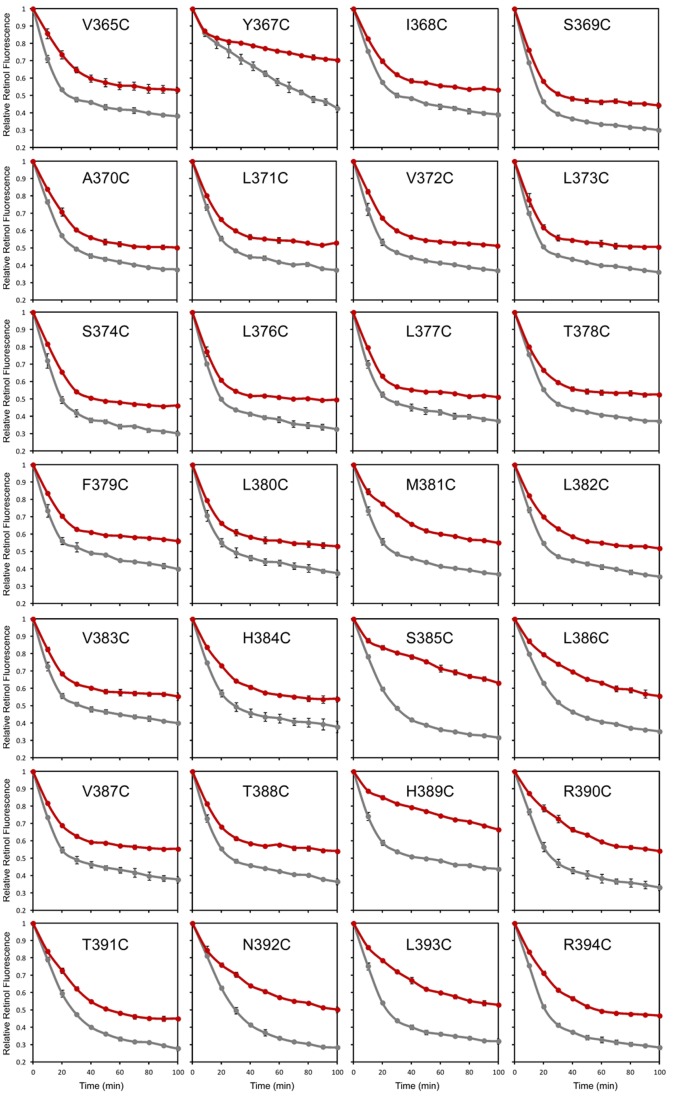
Scanning the seventh transmembrane helix of STRA6 for positions that enhance the sensitivity of STRA6 to MTSEA-biotin modification. Twenty eight residues in or near the seventh transmembrane domain of STRA6 were each changed to cysteine. Each mutant was tested for STRA6-catalyzed retinol release from holo-RBP. Grey traces, no modification. Red traces, MTSEA-biotin modification. All experiments were done in triplicate.

**Figure 6:**
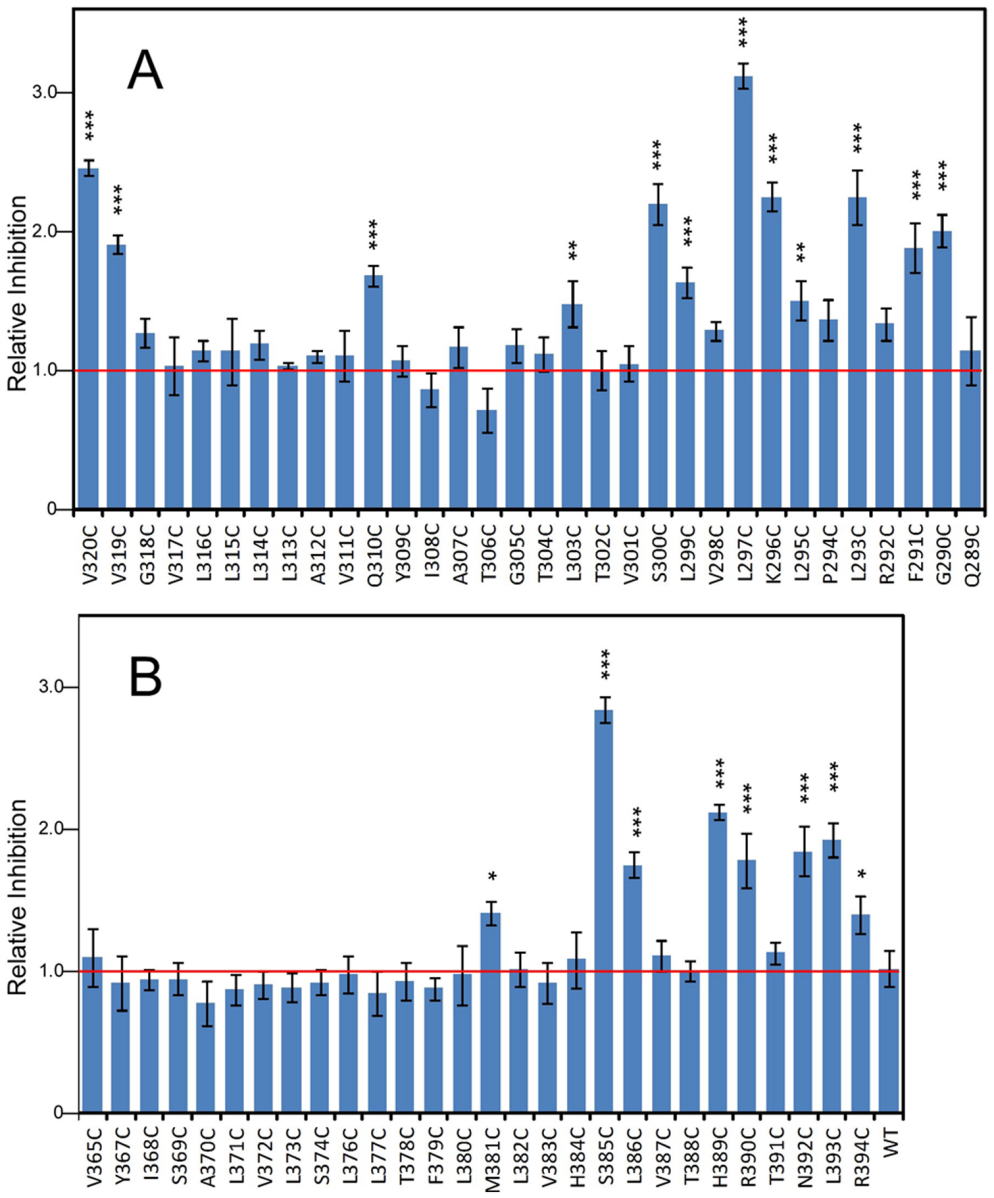
Comparison and quantitation of the acute suppression by MTSEA-biotin for all STRA6 cysteine mutants produced and analyzed in the study. Suppression of the wild-type control is defined as 1. **A.** Mutations in or near the sixth transmembrane helix. **B.** Mutations in or near the seventh transmembrane helix. Statistical analysis is shown as *** (*p*<0.001), ** (*p*<0.01), or * (*p*<0.05). Mutants not labeled were not statistically different from the wild-type control.

**Figure 7:**
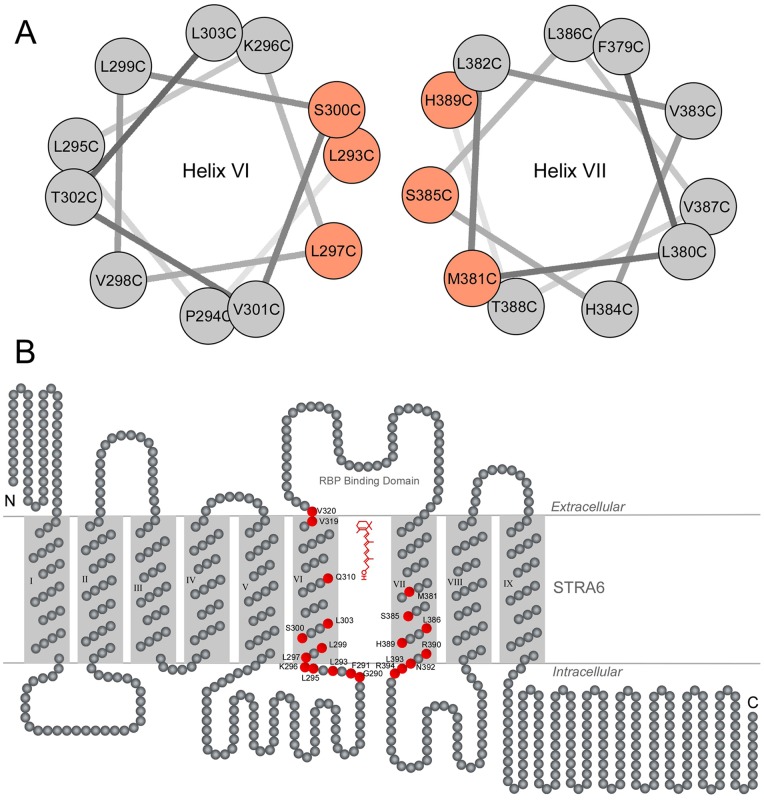
Schematic diagrams of the locations of key positions in STRA6 whose acute modifications block vitamin A transport by STRA6. **A.** Helical wheel presentations of residues L293 to L303 and residues F379 to H389. Residues whose modification by MTSEA-biotin impedes vitamin A transport by STRA6 are labeled in light red. These key residues are located on the same side of the helix. **B.** Locations of the key residues in the transmembrane topology model of STRA6. Residues whose acute modification impedes vitamin A transport by STRA6 are represented as red circles.

We further systematically scanned transmembrane helices VI and VII and adjacent regions by introducing cysteine to each residue. We chose to target these transmembrane helices in this study due to their immediate adjacency to the essential RBP binding domain of STRA6 [47]. We tested each STRA6 mutant for acute chemical modification (Figures 4 and 5). We found that most cysteine mutants behave similarly as the wild-type protein without cysteine modification (Figures 4 and 5). However, cysteine modification of a few of them highly suppresses STRA6′s retinol release activity. From the 60 mutants we created, we identified 20 positions whose modification can significantly suppress STRA6-mediated vitamin A transport (Figure 6). Interestingly, in helix wheel presentations, many key residues on the sixth or the seventh transmembrane helices are located on the same side of the helix (Figure 7A). As shown in the STRA6 transmembrane topology model (Figure 7B), many of the key residues are also located near the cytoplasmic side of STRA6 and are far away from the extracellular RBP binding domain [47].

## Discussion

The RBP receptor is a new type of cell-surface receptor, and the structural basis of this receptor's substrate transport is unknown due to its recent discovery and the technical challenges in studying it. Recently, a STRA6 homolog has been identified which is expressed in liver, intestines and adipose tissue of obese mice [66]. A better understanding of these receptors will help to develop techniques to enhance or suppress their activities as a way to treat human diseases. Disruption of retinoid homeostasis is associated with a variety of human diseases. Unlike other classes of receptors such as G-protein coupled receptors, the RBP receptor STRA6 has 9 transmembrane domains, but the functions of these transmembrane domains were completely unknown.

One strategy to study membrane transport is to use natural blockers of transport. Natural blockers can offer unique insights into transport mechanisms (e.g., tetrodotoxin for sodium channel [67] or charybdotoxin for potassium channel [52]). However, there is no known chemical or toxin that can block vitamin A uptake by the RBP receptor. In addition, vitamin A transport may be theoretically harder to block than the substrates of ion channels because unlike ions, whose movement can be strongly influenced by electrostatic interactions (e.g., charge repulsion), vitamin A has no charge and is much less likely to be influenced by introduced charges. To overcome these difficulties, we employed chemical modification, which makes it possible to acutely modify a functional protein in a site-specific manner and allows the introduction of a wide variety of side chains that are not possible by mutagenesis alone. Using a combination of different techniques including classic radioactivity-based and HPLC-based technique and newly developed real-time monitoring techniques, we showed here that chemical modification of specific positions in the transmembrane domains of STRA6 can block its vitamin A transport. By screening chemical reagents of different sizes and charges and systematically scanning the residues in or near transmembrane helices VI and VII of STRA6, we identified several key residues in STRA6 that are aligned in the direct path of vitamin A transport by STRA6. These blocking effects on vitamin A transport are both highly site specific and chemical modification specific.

As a new type of cell-surface receptor, STRA6 is different from previously known cell-surface receptors that also function as channels/transporters. For example, for the acetylcholine receptor, which is an ion channel, the receptor's ligand acetylcholine is physically distinct from its transport substrate ions [68]. In contrast, the RBP receptor's ligand (RBP) and its transport substrate vitamin A are physically bound together. Experiments presented here suggest that STRA6′s functions go beyond binding RBP and releasing vitamin A from RBP. Since free vitamin A can diffuse through membranes by itself, it was not clear whether vitamin A released from RBP passes through the plasma membrane by random diffusion or through a defined pathway within STRA6. Here, we provide the first experimental evidence that STRA6 has a putative transmembrane pore that interacts with vitamin A during its passage through STRA6. As a hydrophobic chemical, vitamin A may also slide between STRA6′s transmembrane helices to diffuse into the lipid bilayers during STRA6-mediated transport. An interesting finding of this study that argues against this possibility is that many key residues in STRA6 whose modifications block vitamin A transport are located near the intracellular loops. This information suggests that the defined path of vitamin A transport within STRA6 goes as far as intracellular loops and that STRA6 controls vitamin A movement all the way to the intracellular side. The defined pathway for vitamin A transport within STRA6 (as opposed to random diffusion) offers the possibility of regulating its transport, analogous to the gating of ion channels by controlling their pores [51], [52], [53], [54], [55], [56], [57], [68].

## Supporting Information

Figure S1 **Unbiased mutagenesis of STRA6 to reveal mutants defective in vitamin A uptake. Vitamin A uptake activities of random STRA6 mutants 1–840.**
(PDF)

Figure S2 **Unbiased mutagenesis of STRA6 to reveal mutants defective in vitamin A uptake. Vitamin A uptake activities of random STRA6 mutants 841–1680.**
(PDF)

Figure S3 Unbiased mutagenesis of STRA6 to reveal mutants defective in vitamin A uptake. Vitamin A uptake activities of random STRA6 mutants 1681–2520.
(PDF)

Figure S4 Unbiased mutagenesis of STRA6 to reveal mutants defective in vitamin A uptake. Vitamin A uptake activities of random STRA6 mutants 2521–3326.
(PDF)
