# Supplementary material for: Vitamin A Transport and the Transmembrane Pore in the Cell-Surface Receptor for Plasma Retinol Binding Protein
Source: PLoS One. 2013 Nov 1;8(11):e73838. doi: 10.1371/journal.pone.0073838 (PMC3815300; doi:10.1371/journal.pone.0073838)
Supplement: Figure S1 — Unbiased mutagenesis of STRA6 to reveal mutants defective in vitamin A uptake. Vitamin A uptake activities of random STRA6 mutants 1–840. (PDF) [file pone.0073838.s001.pdf]

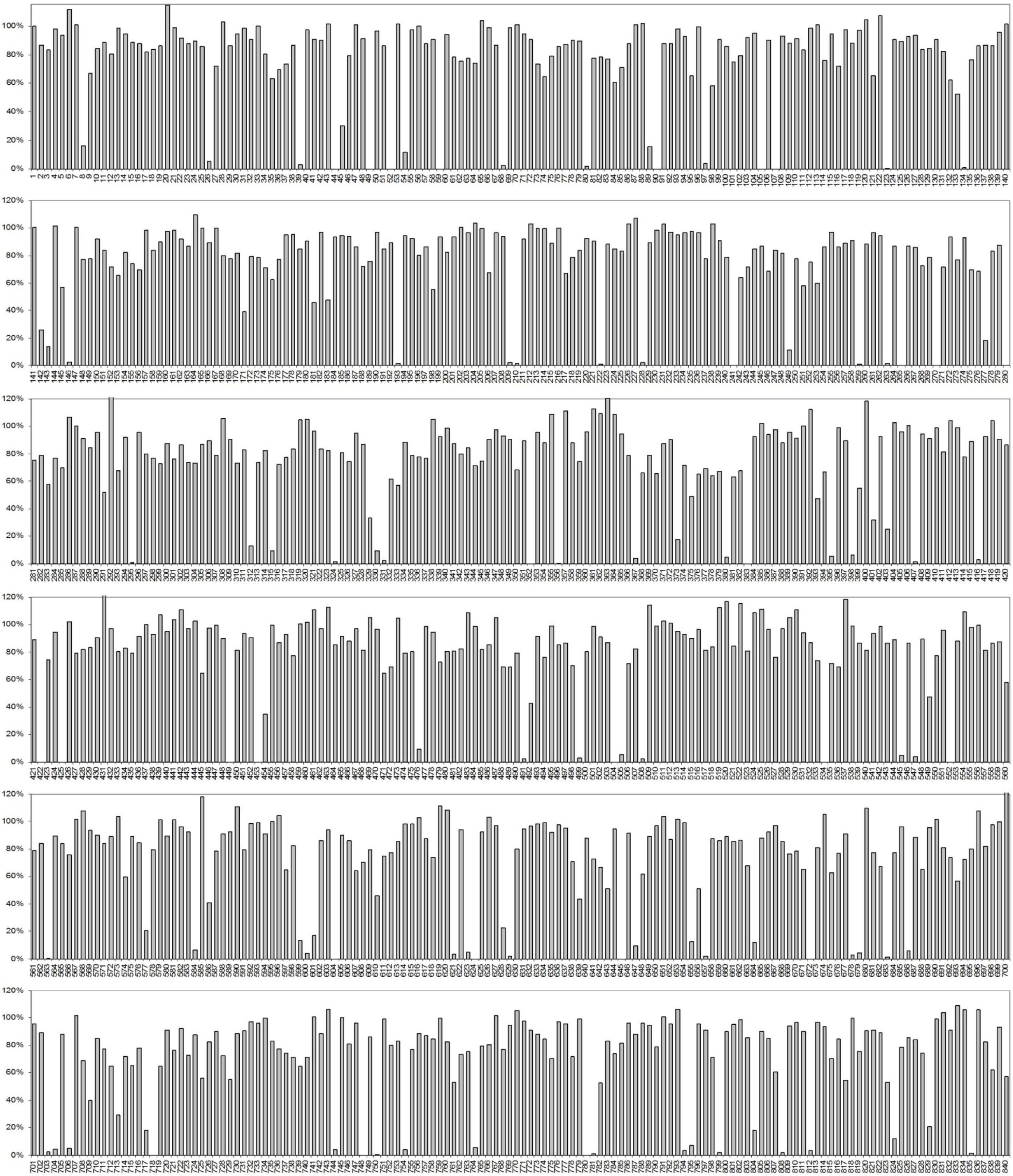

**Figure S1.** Unbiased mutagenesis of STRA6 to reveal mutants defective in vitamin A uptake. Vitamin A uptake activities of random STRA6 mutants 1-840. Activity of wild-type STRA6 is defined as 100%.
